# An innovative approach to predict immune-associated genes mutually targeted by cow and human milk microRNAs expression profiles

**DOI:** 10.14202/vetworld.2018.1203-1209

**Published:** 2018-09-01

**Authors:** Kaj Chokeshaiusaha, Thanida Sananmuang, Denis Puthier, Catherine Nguyen

**Affiliations:** 1 Department of Veterinary Science, Faculty of Veterinary Medicine, Rajamangala University of Technology Tawan-OK, Chonburi, Thailand; 2 Aix-Marseille Université, INSERM UMR 1090, TAGC, Marseille, France

**Keywords:** immune-associated target gene, microRNAs, milk, miRNome

## Abstract

**Aim:**

Milk is rich in miRNAs - the endogenous small non-coding RNA responsible for gene post-transcriptional silencing. Milk miRNAs were previously evidenced to affect consumer’s immune response. While most studies relied on a few well-characterized milk miRNAs to relate their immunoregulatory roles on target genes among mammals, this study introduced a procedure to predict the target genes based on overall milk miRNA expression profiles - the miRNome data of cow and human.

**Materials and Methods:**

Cow and human milk miRNome expression datasets of cow and human milk lipids at 2, 4, and 6 months of lactation periods were preprocessed and predicted for their target genes using TargetScanHuman. Enrichment analysis was performed using target genes to extract the immune-associated gene ontology (GO) terms shared between the two species. The genes within these terms with more than 50 different miRNAs of each species targeting were selected and reviewed for their immunological functions.

**Results:**

A total of 146 and 129 miRNAs were identified in cow and human milk with several miRNAs reproduced from other previous reports. Enrichment analysis revealed nine immune-related GO terms shared between cow and human (adjusted p≤0.01). There were 14 genes related to these terms with more than 50 miRNA genes of each species targeting them. These genes were evidenced for their major roles in lymphocyte stimulation and differentiation.

**Conclusion:**

A novel procedure to determine mutual immune-associated genes targeted by milk miRNAs was demonstrated using cow and human milk miRNome data. As far as we know, this was the 1st time that milk miRNA target genes had been identified based on such cross-species approach. Hopefully, the introduced strategy should hereby facilitate a variety of cross-species miRNA studies in the future.

## Introduction

Milk contains essential nutrients and bioactive components distributed among its three fractions - lipid, whey, and cell. Besides, milk is also an enriched source of non-coding RNAs, especially the microRNAs (miRNAs) which were verified to be absorbed by various consumers’ cells [1-5]. miRNA is an endogenous small non-coding RNA (≈22 nucleotides) responsible for silencing its target messenger RNAs (mRNAs) by post-transcriptional degradation [6] and thus is involved in a variety of cell biological processes [7]. In several breastfeeding animals, mammary gland tissues evidently sequestrate remarkable numbers of miRNAs into milk which, in turn, can modulate particular cell mechanisms of neonate [1-3,5]. Among the whole miRNA spectrum presented - the miRNome expression profiles achieved in milk, the miRNome of milk lipid fraction was regarded to be the best representative for that of mammary gland [1,2].

Equivalent biological influences of milk miRNAs among breastfeeding species were implied by several studies [1-5]. Furthermore, cross-species milk miRNA absorption between human and another breastfeeding mammal was even demonstrated [8,9]. Considering such mutual effects of milk miRNAs, the insight into such conserved orthologous genes should render us with novel knowledge for both animal evolution and pediatric sciences. Since milk miRNome analyses are most conventional among cow and human miRNA studies, miRNome expression profiles of these two species are hence considerably available in the public database - making the cross-species analyses between them priorly suitable and profitable.

Milk miRNAs significantly contributed their roles in neonatal immune system [6,10]. Human milk miRNAs were evidenced to manifest their effects on T-cell function [6,11,12] and B-cell differentiation [6]. Similar immunoregulatory effects were also implied by cow milk miRNAs [10] with reported cross-species effects on human leukocytes [8,9]. These evidence strongly suggested the immune-associated genes mutually targeted by both cow and human milk miRNAs and therefore manifested the conserved immunomodulatory effects of milk miRNAs between the two species. By implying such knowledge to other breastfeeding species, identification of these target genes could beneficially provide us with precious clues into fundamental features of miRNA-mRNA interactions across mammalian evolution [13,14].

Due to the aforementioned necessity, we, therefore, demonstrated a cross-species analytical procedure to predict immune-associated genes mutually regulated by both cow and human milk miRNAs. Based on the cumulative number of miRNAs targeting the genes, the mutual immune-associated genes - functioning in lymphocyte development and resting cell state were successfully demonstrated.

## Materials and Methods

### Ethical approval

All datasets used in this study were available in NCBI SRA public site, and no ethical approval was required.

### Sample datasets

Cow and human milk miRNome expression datasets were acquired from milk lipids of nursing mothers and dairy cows at approximately 2, 4, and 6 months of lactation periods. All cow and human milk miRNome expression datasets with their corresponding sample filename IDs used in this study were retrieved from the sequence read archive (SRA) database (https://www.ncbi.nlm.nih.gov/sra) (Table-1). Further details of each sample file could be attained from SRA database.

**Table-1:** Milk miRNA-seq datasets used in this study.

Species	Description	Sample files
*Bos taurus* (Dairy cow)	Dataset of milk lipid miRNome sequencing approximately at 2 months of lactation	SRR4030778; SRR4030787; SRR4030792 SRR4030797; SRR4030834; SRR4030843 SRR4030852; SRR4030860; SRR4030867
	Dataset of milk lipid miRNome sequencing approximately at 4 months of lactation	SRR4030771; SRR4030779; SRR4030788 SRR4030798; SRR4030831; SRR4030835 SRR4030844; SRR4030861
	Dataset of milk lipid miRNome sequencing approximately at 6 months of lactation	SRR4030772; SRR4030780; SRR4030789 SRR4030799; SRR4030836; SRR4030842 SRR4030845; SRR4030854; SRR4030862
*Homo sapiens* (nursing mother)	Dataset of milk lipid miRNome sequencing approximately at 2 months of lactation	SRR2976292; SRR2976301; SRR2976310 SRR2976316; SRR2976322
	Dataset of milk lipid miRNome sequencing approximately at 4 months of lactation	SRR2976293; SRR2976302; SRR2976311 SRR2976317; SRR2976323
	Dataset of milk lipid miRNome sequencing approximately at 6 months of lactation	SRR2976294; SRR2976303; SRR2976312 SRR2976318; SRR2976324

miRNA=MicroRNAs

### Data preprocessing

The miRNome expression datasets were extracted and checked for sequencing quality as previously described by Chokeshaiusaha *et al*. [15]. Nucleotide sequences of annotated non-coding RNAs applied in this study were retrieved from the Ensembl database (https://asia.ensembl.org/info/data/ftp/index.html). Quality trimming and adapter trimming were performed by Cutadapt v1.14 program [16]. Only sequencing reads with length between 18 and 30 nucleotides and having Phred score ≥20 for at least 50% bases were selected for genome alignment. Alignment was performed with the selected reads corresponding to the species’ genomes - UMD3.1 for cow and GRCh38 for human. Any sequence reads aligned to rRNA, tRNA, snRNA, snoRNA genes, or acquired more than five positions aligned were discarded. The leftover aligned reads were subsequently aligned to miRNA genes. The duplicated sequences were removed [17] and corrected for GC base bias [18]. HTSeq package (version 0.9.1) was applied for miRNA counts [15], and regularized logarithm transformations were performed with the count datasets [19].

### miRNA target genes and gene-annotation enrichment analysis

**Figure-1:**
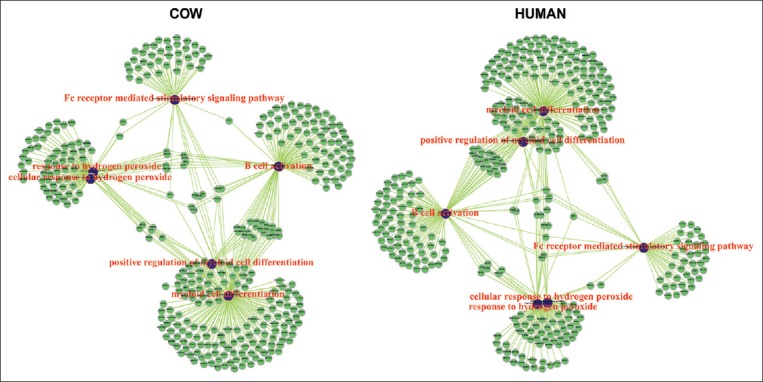
Networks of immune-related gene ontology (GO) terms (•) and target genes of milk microRNAs (•) of cow and human. The presented GO terms were as follows: Response to hydrogen peroxide, myeloid cell differentiation, Fc receptor mediated stimulatory signaling pathway, cellular response to hydrogen peroxide, positive regulation of myeloid cell differentiation, and B-cell activation.

High-confidence miRNA target genes were selected according to their cumulative weighted context scores of the sites (context score ≤0.4 and percentile ≥85), or aggregate probability of conserved targeting ≥0.8 from TargetScanHuman (release 7.1). Enrichment analysis was subsequently performed using target gene list acquired from each species, and significant gene ontology (GO) terms were identified (adjusted p≤0.01) using “clusterprofiler” package [20]. The immune-related GO terms were extracted and observed (Figure-1). miRNA target genes presented in these immune-related GO terms were then determined for the number of miRNA genes targeting them. The genes with more than 50 different miRNA genes acquired from each of species were selected (Table-2) and reviewed for their immunological functions.

**Table-2:** Selected immune-associated genes targeted by cow and human milk miRNAs.

Gene name	Human Ensembl ID	Cow Ensembl ID	Number of miRNA genes targeting	

			Cow	Human
TFRC	ENSG00000072274	ENSBTAG00000032719	60	70
HIF1A	ENSG00000100644	ENSBTAG00000020935	60	68
CREB1	ENSG00000118260	ENSBTAG00000005474	68	100
MYB	ENSG00000118513	ENSBTAG00000012074	52	56
INHBA	ENSG00000122641	ENSBTAG00000002912	93	132
ETS1	ENSG00000134954	ENSBTAG00000002341	172	144
KLF4	ENSG00000136826	ENSBTAG00000020355	76	78
PIK3R1	ENSG00000145675	ENSBTAG00000010989	93	96
PURB	ENSG00000146676	ENSBTAG00000046625	51	63
KLF10	ENSG00000155090	ENSBTAG00000014396	50	68
PPARGC1B	ENSG00000155846	ENSBTAG00000012943	76	126
CRK	ENSG00000167193	ENSBTAG00000005665	110	128
BCL2	ENSG00000171791	ENSBTAG00000019302	84	96
SP3	ENSG00000172845	ENSBTAG00000000176	50	90

miRNA=MicroRNAs

### Data virtualization

**Figure-2:**
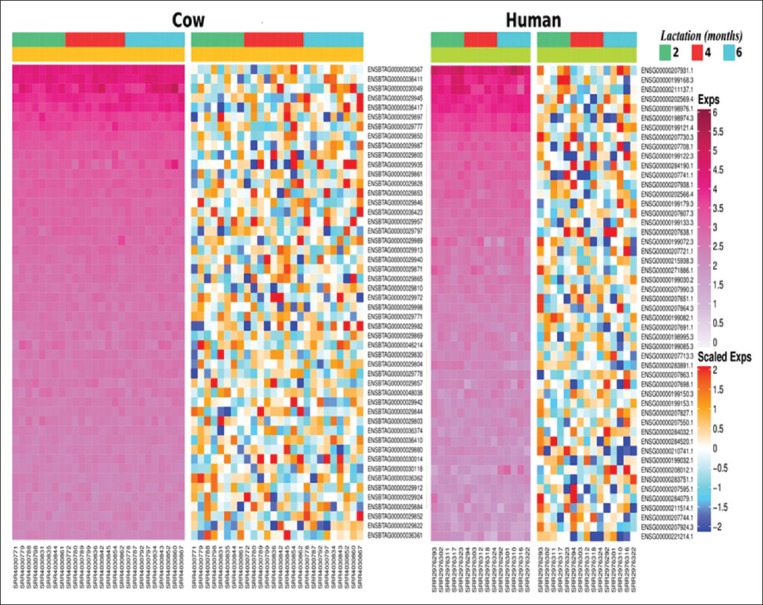
Heatmaps of top 50 cow (cow) and human (human) milk microRNAs read count number in non-scaled regularized logarithm (exps) and scaled regularized logarithm (scaled exps) at 2 (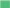), 4 (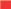), and 6 (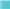) months of lactation ordering by mean expression values.

**Figure-3:**
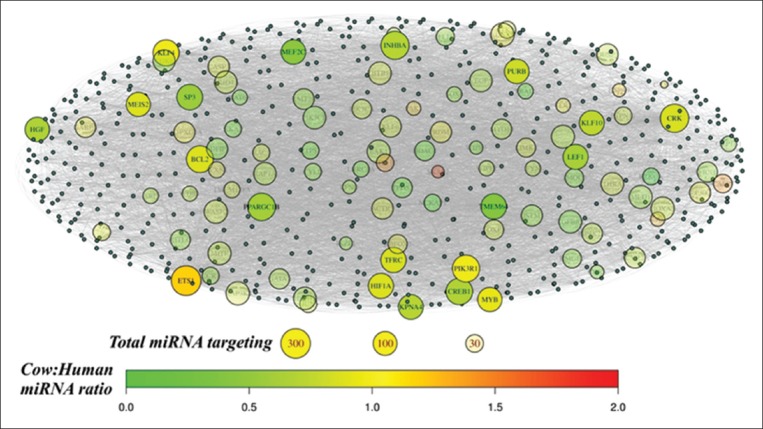
Spherical network of milk microRNAs (miRNA) target genes presented in immune-related gene ontology (GO) terms. Target genes of milk miRNA presented in immune-related GO terms were illustrated in the figure by the number of miRNA targeting them (Total miRNA targeting) indicating by circle size, and also the ratio of cow to human miRNA targeting them scaling from green to red colors (cow: Human miRNA ratio). The target genes with <50 miRNA genes acquired from each of species targeting them (<50 cow miRNAs and <50 human miRNAs) were transparent and were not reviewed for their functions. Each small gray dots represented miRNA without label.

Heatmap of non-scaled and scaled normalized miRNA read count data was drawn by “ComplexHeatmap” package [21] to display miRNAs presented in cow and human milk datasets (Figure-2). To visualize association among the immune-related GO terms and their identified target genes, the network among terms and target genes was plotted using “igraph” package [22] (Figure-1). To illustrate the selection of immune-associated genes targeted by a number of different miRNA genes, the spherical interacting network among them was plotted (Figure-3).

## Results and Discussion

### Cow and human milk miRNAs

To acquire the results that represented mammary gland functions in miRNA productions during mid-lactation period of both cow and human, this study adopted milk lipid miRNome expression profiles during 3-6 months of lactational periods acquired from both species. A total of 146 and 129 miRNAs were identified in cow and human milk, accordingly. Supporting the previous study [2], similar miRNA expressions among three lactation periods were demonstrated (non-scaled heat map of Figure-2). Top 50 expressed miRNAs ranked by mean expression values were presented (Table-3), by which were concordant with other previous reports [1,6,10]. Interestingly, some miRNAs with immunoregulatory functions were also included (Table-3). For example, bta-mir-142 (ENSBTAG00000029982) [23], bta-mir-146a (ENSBTAG00000029830) [24], and bta-mir-148b (ENSBTAG00000029852) [25] were evidenced for their immunosuppressive functions in cow. Similarly, Hsa-mir-181a-2 (ENSG00000207595.1), Hsa-mir-125b-2 (ENSG00000207863.1), Hsa-mir-146b (ENSG00000202569.4), and Hsa-let-7i were well-recognized members of immunosuppressive miRNAs in human [26-29].

**Table-3:** Top 50 miRNA genes presented in cow and human milk.

Organisms	Top 50 miRNAs
Cow	ENSBTAG00000036367, ENSBTAG00000036411, ENSBTAG00000030049, ENSBTAG00000029945, ENSBTAG00000036417, ENSBTAG00000029897, ENSBTAG00000029777, ENSBTAG00000029850, ENSBTAG00000029987 ENSBTAG00000029800, ENSBTAG00000029935, ENSBTAG00000029861, ENSBTAG00000029828, ENSBTAG00000029853, ENSBTAG00000029846, ENSBTAG00000036423, ENSBTAG00000029957, ENSBTAG00000029797 ENSBTAG00000029989, ENSBTAG00000029913, ENSBTAG00000029940, ENSBTAG00000029871, ENSBTAG00000029865, ENSBTAG00000029810 ENSBTAG00000029972, ENSBTAG00000029998, ENSBTAG00000029771 ENSBTAG00000029982, ENSBTAG00000029869, ENSBTAG00000046214 ENSBTAG00000029830, ENSBTAG00000029804, ENSBTAG00000029778 ENSBTAG00000029857, ENSBTAG00000048038, ENSBTAG00000029942 ENSBTAG00000029844, ENSBTAG00000029803, ENSBTAG00000036374 ENSBTAG00000036410, ENSBTAG00000029880, ENSBTAG00000030014 ENSBTAG00000030118, ENSBTAG00000036362, ENSBTAG00000029912, ENSBTAG00000029924, ENSBTAG00000029884, ENSBTAG00000029852 ENSBTAG00000029822, ENSBTAG00000036361
Human	ENSG00000207931.1, ENSG00000199168.3, ENSG00000211137.1 ENSG00000202569.4, ENSG00000198976.1, ENSG00000198974.3 ENSG00000199121.4, ENSG00000207730.3, ENSG00000207708.1 ENSG00000199122.3, ENSG00000284190.1, ENSG00000207741.1 ENSG00000207938.1, ENSG00000202566.4, ENSG00000199179.3 ENSG00000207607.3, ENSG00000199133.3, ENSG00000207638.1 ENSG00000199072.3, ENSG00000207721.1, ENSG00000215938.3 ENSG00000271886.1, ENSG00000199030.2, ENSG00000207990.3 ENSG00000207651.1, ENSG00000207864.3, ENSG00000199082.1 ENSG00000207691.1, ENSG00000198995.3, ENSG00000199085.3 ENSG00000207713.3, ENSG00000283891.1, ENSG00000207863.1 ENSG00000207698.1, ENSG00000199150.3, ENSG00000199153.1 ENSG00000207827.1, ENSG00000207550.1, ENSG00000284032.1 ENSG00000284520.1, ENSG00000210741.1, ENSG00000199032.1 ENSG00000208012.1, ENSG00000283751.1, ENSG00000207595.1 ENSG00000284079.1, ENSG00000211514.1, ENSG00000207744.1 ENSG00000207924.3, ENSG00000221214.1

miRNA=MicroRNAs

### Enrichment analysis and prediction of mutual immune-associated target genes of milk miRNAs

Prediction of milk miRNA target genes was performed by TargetScanHuman. We considered TargetScanHuman suitable for our study due to its principle in comparison of conserved 3’UTR among orthologous sequences of human and other mammalian transcripts including cow [14,30]. All predicted target genes were authorized for gene-annotation enrichment analysis, and several terms associated developmental processes and cell mechanisms were signified (adjusted p≤0.01) concordant with other previous reports [1,2]. Among them, nine terms were biologically immune-related and also shared between cow and human - implying them as the conserved miRNAs regulating processes. These terms were as follows: Response to hydrogen peroxide, myeloid cell differentiation, Fc receptor mediated stimulatory signaling pathway, cellular response to hydrogen peroxide, positive regulation of myeloid cell differentiation, and B-cell activation (Figure-1). The genes related to these terms were then determined for cow and human miRNAs targeting them. The ratio of cow to human miRNAs targeting each gene revealed that most genes were targeted by cow more than human miRNAs (Cow: Human miRNA ratio >1 in Figure-3). Among them, 14 genes were featured with more than 50 miRNA genes of each species targeting them (Figure-3 and Table-2) including the top expressed ones (Table-1).

As mentioned in the introduction, lymphocytes were implied as common immune cells affected by intake milk miRNA [6,8-12]. Concordant with other previous reports [6,9-11], our acquired mutual immune-associated genes also manifested their roles with lymphocyte differentiation, activation, and proliferation. Post-transcriptional gene silencing of milk miRNAs on them should thus contribute to modulation of lymphocyte development and also maintaining a non-activating state of lymphocytes of which would be reviewed later. While most previous studies attributed regulatory contributions of milk miRNAs to some well-characterized miRNAs, such assumptions could overlook several other genes significantly targeted by the whole redundant miRNAs presented in milk. In this study, prediction of candidate genes iteratively targeted by overall miRNAs (≥50 miRNAs of each species) should provide an additional strategy to determine the immunoregulatory function of milk miRNAs on such genes.

### Immune-associated genes iteratively targeted by cow and human milk miRNAs

Immunoregulatory functions of the acquired 14 genes (Table-2) were briefly reviewed as follows. Expressions of CREB, EST1, KLF4 MYB, PPARGC1B, and TFRC were related to increases in lymphocyte activation, lymphocyte proliferation, and inflammation [31-38]. PURB encoded a transcription activator of MYC molecule - a key transcription factor required during dendritic cell (DC) maturation and lymphocyte activation [39]. HIF1A - a subunit gene of HIF-1 transcription factor could activate DC and inhibit the regulatory function of T-cell simultaneously [40]. INHBA encoded a subunit of activin - a protein required for several pro-inflammatory cytokine releases [41]. PIK3R1 encoded the regulatory subunit 1 of PI3K - a kinase adapter protein required for lymphoid/myeloid cell development and functions [42]. CRK encodes an adapter protein participating in several cell signaling pathways, while BCL2 encoded a universal antiapoptotic molecule commonly upregulated during lymphocyte development and activation [43,44]. SP3 was crucial for expressions of some surface molecules and cytokines in lymphocytes and myeloid cells [45,46]. Among all predicted target genes, KLF10 was the only gene with a suppressive function on T-cell proliferation [47,48]. While the effective evaluation system for overall milk miRNAs’ regulation on target leukocyte was still undetermined, these target genes could become potential candidates for such studies in the future.

## Conclusion

The current study demonstrated a novel procedure to determine immunoregulatory genes comprehensively targeted by cow and human milk miRNAs. The acquired candidate genes - based on immune-related GO terms strongly suggested the plausible roles of milk miRNAs in maintaining the cell’s homeostasis and its resting state. With validated determination system, these candidate genes should hereby establish numbers of further interesting novel research topics. Of note, this study aimed to demonstrate a beneficial strategy for future cross-species miRNA target genes employing immune system for demonstration - by which was able to apply with other biological systems of interest.

## Authors’ Contributions

KC and TS planned the study design, collected the datasets and analyzed the data. CN refined the study design and the objective. DP carried out technical coding correction and hardware maintenance. KS and TS rafted and reviewed the manuscript. All authors read and approved the final manuscript.
